# Kyste hydatique à localisation costo vertébrale

**DOI:** 10.11604/pamj.2014.19.343.5478

**Published:** 2014-12-02

**Authors:** Rachid Marouf

**Affiliations:** 1 Service de Chirurgie Thoracique, CHU Mohammed VI, Oujda, Maroc

**Keywords:** Hydatidose osseuse, hydatidose costo-vertébrale-diagnostic, chirurgie, bone hydatid, costo-vertebral hydatidosis diagnosis, surgery

## Abstract

L'hydatidose est une affection parasitaire due à la contamination de l'homme par la forme larvaire de ténia échinococcus granulosus, la forme costo vertébrale est une localisation très rare qui représente 0,18 à 1,21% de l'ensemble des localisations hydatiques. Nous rapportons le cas d'une femme de 32 ans qui présente un kyste hydatique multi vésiculaire à localisation costovertébrale, traité par chirurgie radicale associée à un traitement médical anti parasitaire pour une durée de 6 mois, avec bonne évolution. L'atteinte costo-vertébrale par la maladie hydatique est rare et l’évolution est insidieuse. Malgré un traitement chirurgical radical, la fréquence des récidives rend le pronostic sombre.

## Introduction

Malgré l’état endémique de la maladie hydatique dans les pays du Maghreb, l'hydatidose osseuse reste une affection rare. L'hydatidose costo-vertébrale est une localisation exceptionnelle et représente 0,18 à 1,21% [1]. Le diagnostic est souvent tardif par manque de spécificité et latence clinique qui caractérisent cette affection. L'imagerie est essentielle au diagnostic positif et au bilan d'extension vertébrale et aux parties molles. Son évolution est insidieuse et son pronostic est sombre du fait des récidives fréquentes surtout en cas d'exérèse incomplète.

## Patient et observation

Nous rapportons le cas d'une femme de 32 ans, ayant comme antécédent un kyste hydatique du foie opérée il y a 16 ans, qui se présente pour une dyspnée d'effort et des douleurs thoraciques postérieures gauches évoluant depuis 3 mois, sans signes neurologiques associés. L'examen clinique trouve une matité basi-thoracique gauche avec des douleurs exquises à la palpation de cette région.

**Figure 1:**
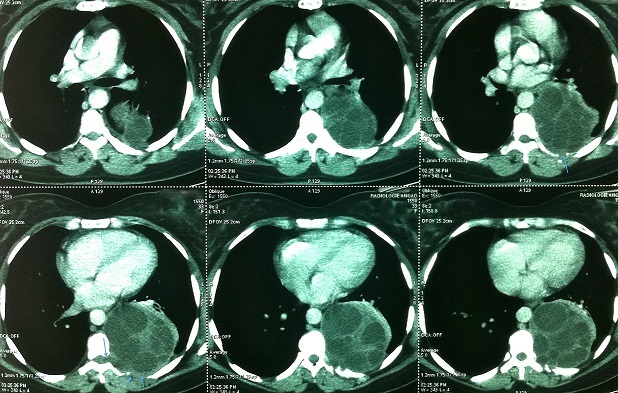
TDM thoracique: aspect de kyste hydatique multi vésiculaire postéro basal gauche, de la gouttière costovertébrale au dépend des 7^ème^ et 8^ème^ côtes avec lyse costale, et des vertèbres correspondantes, et qui s’étend jusqu'au canal rachidien sans signes d'envahissement ou de compression, et sans atteinte abdominale associée

Le scanner thoraco-abdominal avec myélo scanner montre un aspect de kyste hydatique multi vésiculaire postéro basal gauche, de la gouttière costo vertébrale au dépend des 7^ème^ et 8^ème^côtes avec lyse costale, et des vertèbres correspondantes, et qui s’étend jusqu'au canal rachidien sans signes d'envahissement ou de compression, et sans atteinte abdominale associée (Figure 1).

L'imagerie par résonance magnétique (IRM) trouve une formation multi vésiculaire de la gouttière costo vertébrale englobant l'arc postérieur des 7^e^, 8^e^ côtes qui sont lysées avec lyse de l'apophyse transverse de la 7^e^ et de la 8^e^ vertèbres dorsales (T7-T8) et extension intra canalaire dans l'espace épidural T7-T8 sans atteinte médullaire. La sérologie hydatique est positive. Le bilan préopératoire est normal.

**Figure 2:**
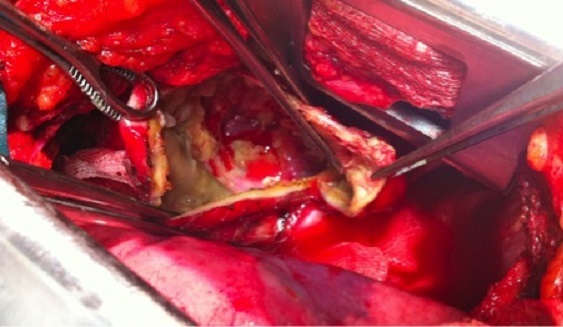
Évacuation du kyste hydatique costo-vertébral avec kystectomie

**Figure 3:**
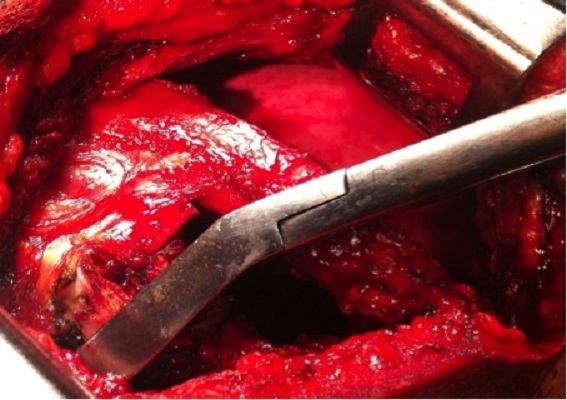
Résection de l'arc postérieur de la 7ème-8ème cotes emportant les apophyses transverses et les muscles para vertébraux en regard

**Figure 4:**
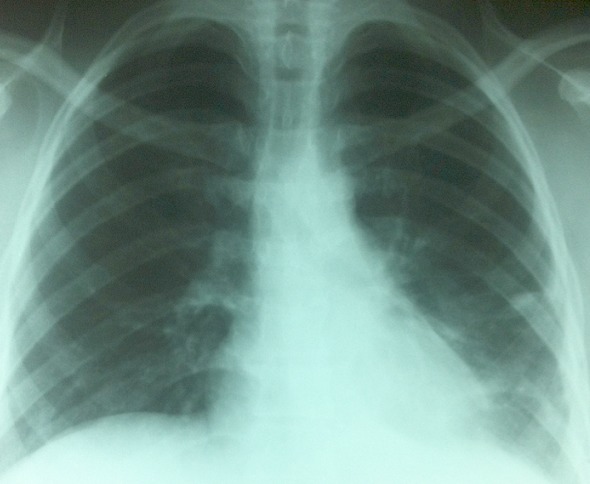
Radiographie thoracique de contrôle après 6 mois de l'intervention

Un traitement chirurgical radical est réalisé: kystectomie avec résection de l'arc postérieur de la 7^ème^- 8^ème^ cotes emportant les apophyses transverses et les muscles para vertébraux en regard et une laminectomie de D7-D8 (Figure 2, Figure 3). Les suites postopératoires sont simples. Un traitement médical anti parasitaire (Albendazole à raison de 800mg/J) pour une durée de 6 mois a été instauré. L’évolution est favorable avec un recul de 12 mois (Figure 4).

## Discussion

L’échinococcose est une anthropozoonose provoquée par la forme larvaire d'un cestode de l'espèce Echinococcus granulosis. L'hydatidose osseuse est rare, elle constitue 0,5 à 2% de l'ensemble des hydatidoses [1]. La contamination osseuse est primitive, rarement secondaire, se faisant par voie hématogène. La localisation vertébrale est la plus grave du fait de l'aspect étendu et complexe des lésions et par la fréquence de l'extension endo canalaire et de l'atteinte neurologique par compression médullaire. Cette forme s'associe souvent à une atteinte costale [2, 3].

La symptomatologie clinique est insidieuse et non spécifique, c'est pourquoi le diagnostic est souvent tardif; elle se traduit par des douleurs thoraciques, une tuméfaction pariétale, une fracture ou des manifestations neurologiques s'exprimant parfois par un syndrome de compression médullo-radillaire témoignant de l'envahissement du canal médullaire. Le diagnostic est basé sur des arguments épidémiologiques, cliniques, biologiques et radiologiques [4–6].

La tomodensitométrie (TDM) en association avec un myélo-scanner est l'examen de référence pour le diagnostic de cette forme costo-vertébrale. Elle permet une analyse fine des lésions osseuses en montrant des lésions multi loculées de densité liquidiennesavec des cloisons fines, une lyse osseuse avec corticale soufflée laminée ou rompue,de localiser le processus expansif, et peutaussi évaluer l'extension osseuse vertébrale, intra-canalaire et aux parties molles des vésicules hydatiques [3, 7].

L'IRM occupe actuellement une place de choix, elle montre des images hydatiques caractéristiques de signal bas sur les séquences pondérées en T1 et de signal élevé sur les séquences pondérées en T2 ne prenant pas le contraste sauf parfois en périphérie [2]. Elle a l'avantage par rapport au TDM d'apprécier les rapports des kystes avec les structures avoisinantes en particulier les tissus mous surtout au rachis et à la moelle épinière [4]. Elle est obligatoire en cas d'atteinte de l'arc postérieur de la vertèbre. Elle permet aussi de juger de la viabilité du kyste qui est suggérée par un hyper signal en T2 alors que la mort du kyste est suspectée devant une diminution de l'intensité du signal [2]. Les tests immunologiques sont positifs au stade d'invasion ou lorsque le kyste hydatique est remanié ou fissuré. Ils ont aussi un rôle dans le suivi post-thérapeutique.

La chirurgie demeure le traitement de choix, elle consiste en une résection large des os atteints et des muscles avoisinants. Malheureusement cette résection est rarement radicale et les récidives sont fréquentes. Le but de cette chirurgie est de lever la compression nerveuse et de reséquer la totalité du kyste hydatique.

La thoracotomie postéro latérale permet un abord de tous les corps vertébraux du rachis dorsal et une résection costale élargie aux muscles et aux vertèbres avec possibilité de reconstruction ou d'ostéosynthèse et permet aussi de traiter une localisation pulmonaire ou médiastinale associée [8].

Un traitement médical anti parasitaire par l'albendazole est préconisé en association avec la chirurgie en pré et post opératoire pour prévenir les récidivesou seul à fortes doses (800mg/j) dans les formes inopérables pour minimiser le risque de dissémination pour une longue durée (6 à 9 mois) [7, 8].

## Conclusion

L'hydatidose costo vertébrale est très rare. Le diagnostic radiologique se base sur Le bilan scannographique et sur l'IRM. La chirurgie est le traitement de choix. Le pronostic est incertain du fait du risque de récidive locale et de compression médullaire.
